# Bacterial extracellular vesicles-based therapeutic strategies for bone and soft tissue tumors therapy

**DOI:** 10.7150/thno.78034

**Published:** 2022-09-11

**Authors:** Han Liu, Hao Zhang, Yafei Han, Yan Hu, Zhen Geng, Jiacan Su

**Affiliations:** 1 Institute of Translational Medicine, Shanghai University, Shanghai, 200444, China.; 2 Musculoskeletal Organoid Research Center, Shanghai University, Shanghai, 200444, China.

**Keywords:** Bone and soft tissue tumors, Bacteria extracellular vesicles, Immunotherapy, Synergistic therapy, Nanotechnology

## Abstract

Bone and soft tissue tumors are complex mesenchymal neoplasms that seriously endanger human health. Over the past decade, the relationship between microorganisms and human health and diseases is getting more attention. The extracellular vesicles derived from bacteria have been shown to regulate bacterial-host cell communication by transferring their contents, including nucleic acids, proteins, metabolites, lipopolysaccharides, and peptidoglycans. Bacteria extracellular vesicles (BEVs) are promising lipid-bilayer nanocarriers for the treatment of many diseases due to their low toxicity, drug loading capacity, ease of modification and industrialization. Specially, BEVs-based cancer therapy has attracted much attention because of their ability to effectively stimulate immune responses. In this review, we provide an overview of the biogenesis, composition, isolation, classification, and internalization of BEVs. We then comprehensively summarize the sources of BEVs in cancer therapy and the BEVs-related cancer treatment strategies. We further highlight the great potential of BEVs in bone and soft tissue tumors. Finally, we conclude the major advantages and challenges of BEVs-based cancer therapy. We believe that the comprehensive understanding of BEVs in the field of cancer therapy will generate innovative solutions to bone and soft tissue tumors and achieve clinical applications.

## Introduction

Bone and soft tissue tumors (BSTTs) account for approximately 1% of adult malignancies and about 20% of pediatric neoplasms 1. Nearly 200,000 people are diagnosed with sarcoma each year in the world 2. As a diverse and heterogeneous group, these tumors comprise more than 50 subtypes, of which approximately half are musculoskeletal tumors occurring in the extremities 3, 4. The distribution of BSTTs sites, mainly including head and neck, skin, trunk, limbs, and other sites. Currently, the main treatment for BSTTs is a combination of surgery and chemotherapy 5. In addition, some therapies such as gene therapy and immunotherapy have achieved certain results 6, 7. However, in the past 30 years, the progress in primary malignancies treatment has remained slow and clinical outcomes have not been significantly improved. Therefore, it is urgent to explore innovative treatment strategies for BSTTs.

The human body is a complex ecosystem inhabited by trillions of microorganisms, such as bacteria, fungi, and viruses 8. The Human Microbiome Project (HMP) supported by National Institutes of Health (NIH) has greatly expanded our understanding of the relationship between the human microbiome and human health and disease. It has been reported that more than 1000 species of microorganisms inhabit in a healthy human body 9. These microorganisms are widely parasitic in the oral cavity, genitourinary tract, skin, and gastrointestinal tract, and affect the human health and disease in a subtle and complex way 10. Recently, increasing evidence has shown a strong link between intestinal dysbiosis and BSTTs 11, 12. Although the mechanism exploration 13-15 and bioactive material development 16-19 of bone and soft tissue diseases have been well developed, the mechanism of commensal bacteria affecting disease still needs further exploration.

**Figure 1:**
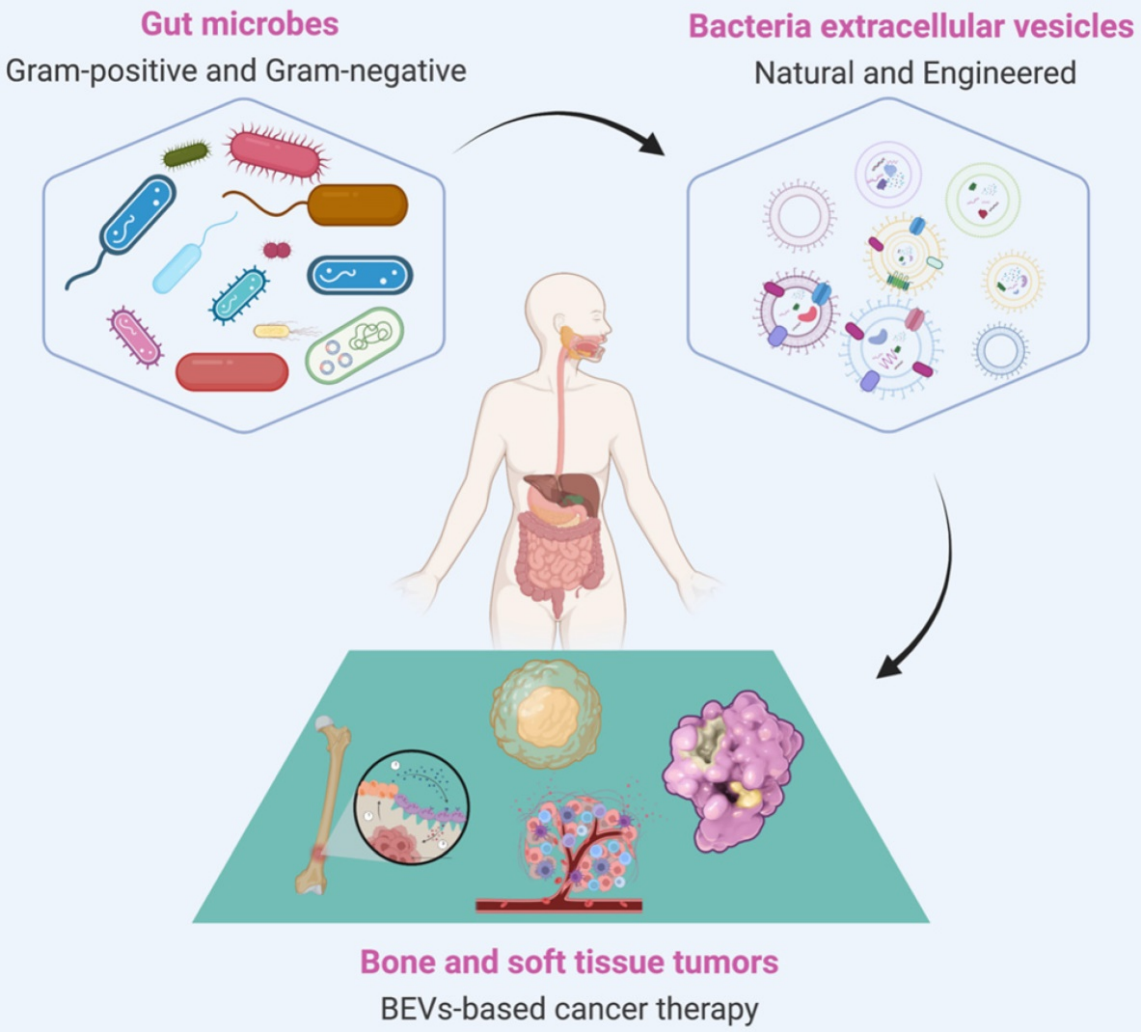
** BEVs-based cancer therapy is of great significance.** BEVs are derived from Gram-positive and Gram-negative bacteria and can be designed as functionalized BEVs for tumor therapy by engineering approaches. The resulting BEVs have shown great promise against various tumors, including bone and soft tissue tumors. Figure was created with https://app.biorender.com/.

Extracellular vesicles (EVs) are particles with lipid bilayer released by all domains of life, including eukaryotes, bacteria, and archaea 20, 21. The relationship between EVs and BSTTs has also been focused 22-24. The growing understanding of human microbial communities in health and disease has led to insights into EVs derived from microorganisms, especially the bacterial EVs (BEVs), and their roles in microbiota-host communication 25, 26. BEVs are thought to modulate intercellular communications by transferring their contents including nucleic acids (DNA, RNA, miRNA), proteins (cytoplasmic and periplasmic proteins), metabolites, lipopolysaccharide (LPS), and peptidoglycan 27, 28. The intersection of microorganisms and EVs is emerging as attractive research in the biomedical field. In recent years, the roles of BEVs in promoting health and causing pathologies are becoming increasingly obvious 29, 30. The growing recognition that BEVs can enter the systemic circulation and be detected in human body fluids, where it may stimulate the progress of microbiome research, liquid biopsies technology, and BEV-based therapies 31. Importantly, bacteria have the advantages of rapid proliferation and mature fed-batch culture technology 32-34, BEVs-based therapy is a promising strategy to overcome large-scale production problems associated with mammalian EVs (MEVs) and other synthetic nanomaterials 35, 36. In addition, advance in synthetic biology have also made it possible to use bioengineered BEVs to precisely deliver effective agents to cancer cells or tissue 37, 38. BEVs have emerged as a new drug delivery vehicle and crucial signaling mediators with great potential for clinical application due to the advantages of nanosized structure, safety, stable loading capacity, good biocompatibility, ease of modification and production 39-41. In conclusion, the development of BEVs and their applications in the treatment of BSTTs is of great significance (**Figure 1**).

In this review, an overview of the biogenesis, composition, isolation, classification, and internalization of BEVs is summarized. Then, special attention is focused on the sources of BEVs in cancer therapy and the BEVs-based cancer treatment strategies. Moreover, the potential role of BEVs in BSTTs is highlighted. Finally, the major advantages and challenges of BEVs in the treatment of cancer are comprehensively discussed.

## Overview of BEVs

In the past decade, BEVs-based cancer therapy has attracted much attention in the biomedical field 31. BEVs are a promising platform for treating and preventing many diseases due to the ability to deliver virulence, transmit genetic material, and regulate signaling pathway 42. For better understand the applications of BEVs in cancer, we summarize the biogenesis, composition, classification, isolation, and internalization of BEVs.

## Biogenesis, composition, and classification of BEVs

**Figure 2:**
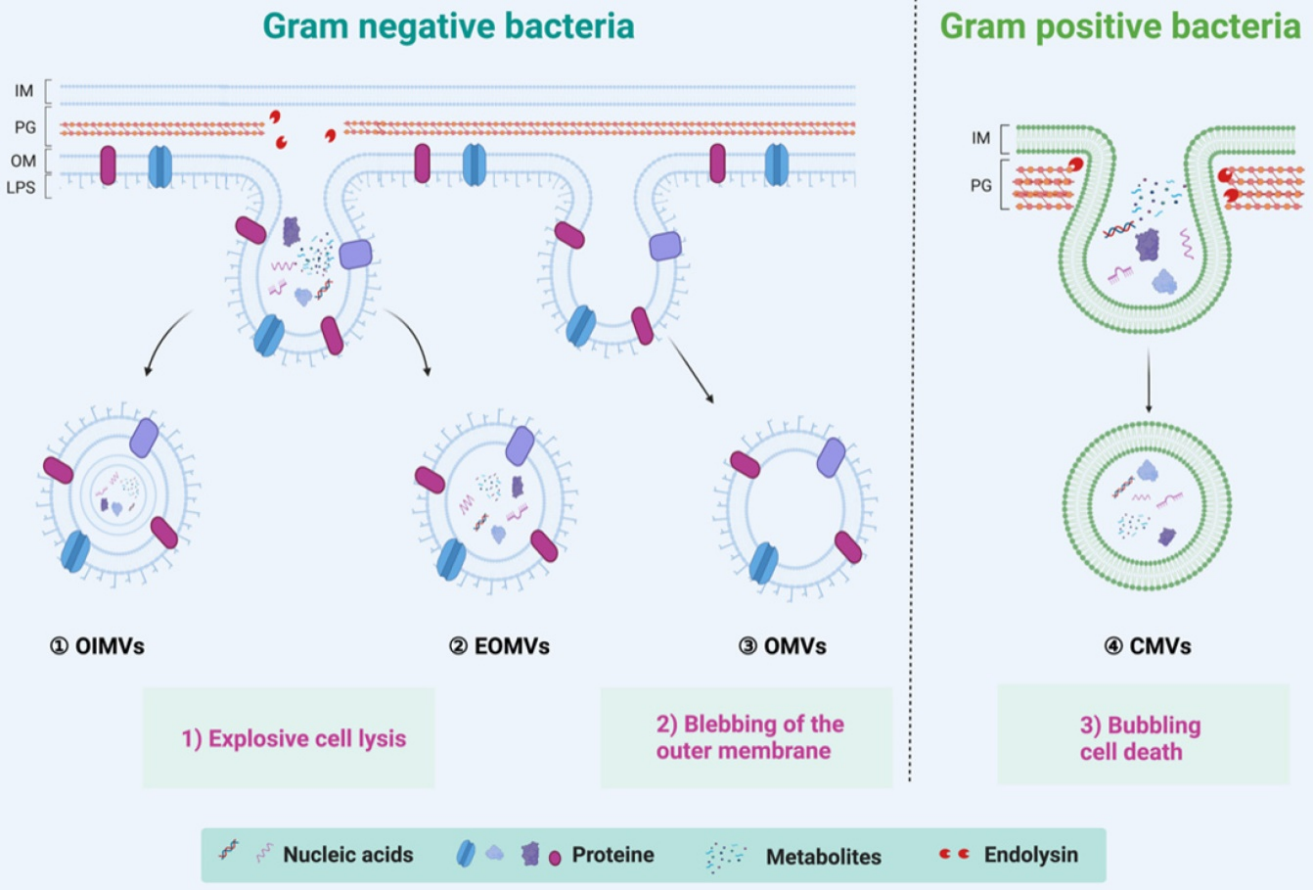
** Overview of biogenesis, composition, and classification of BEVs.** The Gram-positive bacteria can generate CMVs by the mechanism of bubbling cell death. In contrast, the Gram-negative bacteria have two kinds of mechanisms to generate BEVs. The OMVs are produced by the mechanism of blebbing of the outer membrane; the OIMVs and EOMVs are resulted from explosive lysis. In general, BEVs contain many inclusions such as nucleic acids (DNA/RNA), proteins, and metabolites, etc. IM: Inner membrane, OM: other membrane, PG: peptidoglycan, LPS: lipopolysaccharide.

Both commensal and pathogenic bacteria could secrete EVs, a spherical membrane particles with a size of 20~400 nm in diameter 27. Since bacteria can be divided into Gram-positive and Gram-negative bacteria, BEVs are also divided into two categories according to the source of the parental strains 39. The Gram-positive bacteria, such as *lactobacillus rhamnosus*, *staphylococcus aureus*,* diplococcus pneumoniae,* and *Bacillus spp*, were considered incapable of producing BEVs due to its thick peptidoglycan (or thick cell wall) 43. However, growing evidence suggests that Gram-positive bacteria can secrete cytoplasmic membrane vesicles, named CMVs, through a mechanism of bubbling cell death (Right of **Figure 2**) 44. CMVs contain a lot of cargoes, including DNA, RNA, plasmic membrane proteins, virulence factors, and endolysins 45. On the other hand, the Gram-negative bacteria, such as *Escherichia coli*, *Salmonella sp.*, *Helicobacter pylori*, and* Akkermansia muciniphila*, have two kinds of mechanisms to generate outer membrane vesicles (OMVs), outer-inner membrane vesicles (OIMVs), and explosive outer-membrane vesicles (EOMVs) 27 (Left of **Figure 2**). OMVs were generated by blebbing of the outer membrane and thus contain large amounts of outer membrane proteins and lipids 27, 39. The OIMVs and EOMVs were produced by explosive cell lysis and are rich in outer membrane proteins, cytoplasmic (or inner) membrane proteins, plasmids, RNA, DNA, endolysins, virulence factors, and phages 39, 46. Generally, BEVs derived from Gram-negative always contain the innate immune response activator LPS, while Gram-positive BEVs do not contain 47. It's worth pointing out that there are exceptions. *Escherichia coli* Nissle 1917 (EcN) is a very special Gram-negative bacteria that does not contain intact LPS and is often used as a probiotic for the treatment of inflammatory gastrointestinal dysfunction 48. Naturally, ECN-derived EVs do not contain complete LPS. Furthermore, the *msbB* mutant Gram-negative bacteria derived EVs do not contain intact LPS 38, 49.

## Isolation of BEVs

Efficient isolation methods of BEVs determines their further application 50, 51. Many isolation and purification techniques have been developed to obtain high quality BEVs from culture broth. Traditional isolation methods include ultracentrifugation, ultrafiltration, precipitation, affinity isolation, size exclusion chromatography, and density gradient centrifugation 52, 53. The major advantages and disadvantages of these isolation methods have been summarized in Table 1. In general, these methods can obtain great efficiency and purity of BEVs. However, the combination of these methods may achieve better results. For example, Liu et al. 54 collected high purity BEVs derived from *Akkermansia muciniphila* through the combination of ultracentrifugation, ultrafiltration, and density gradient centrifugation.

**Figure 3:**
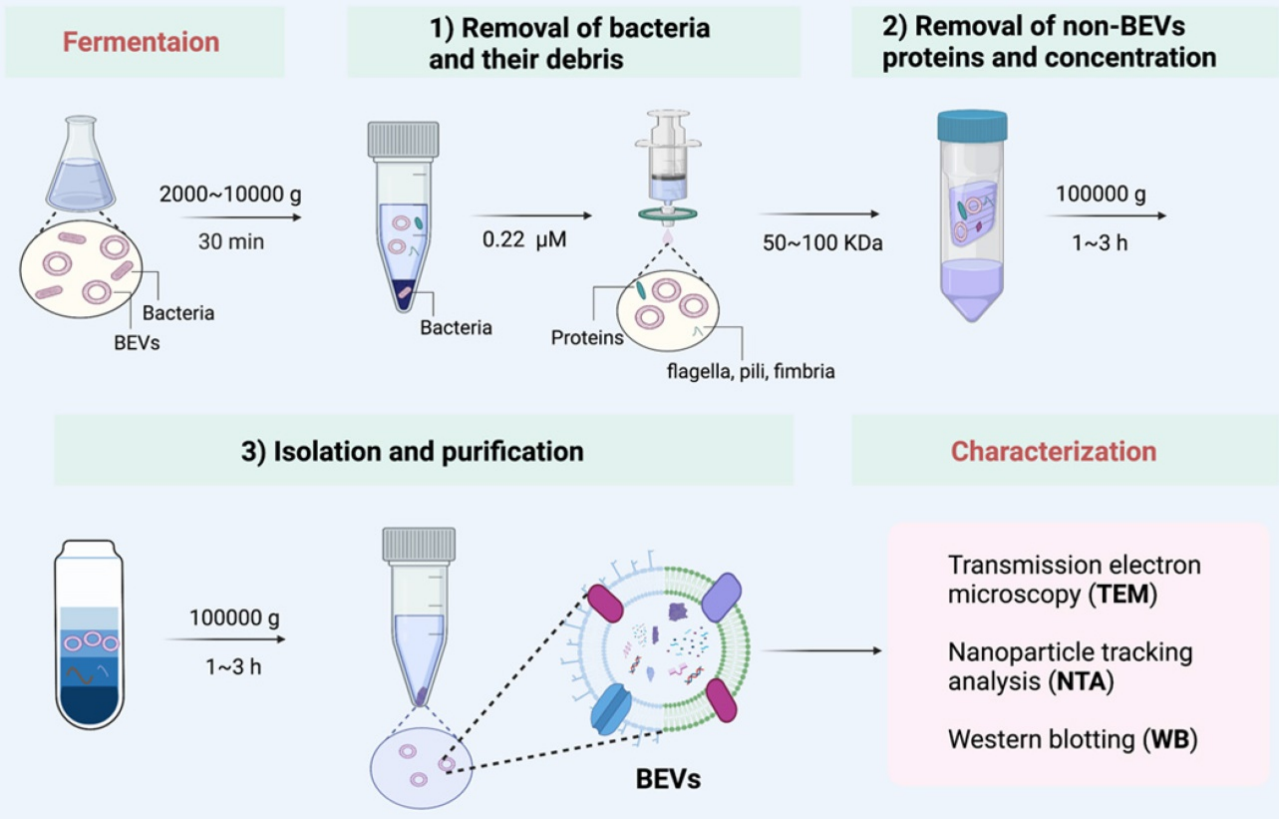
** A set of isolation method applicable to the vast majority of bacteria.** After proper culture, the isolation of BEVs is generally divided into three steps: 1) Removal of bacteria and their debris; 2) Removal of non-BEVs proteins and concentration; 3) Isolation and purification. Finally, the collected BEVs are characterized by TEM, NTA, and WB, if necessary.

Here, we summarized a set of isolation method applicable to the vast majority of bacteria (**Figure 3**). We used this method to successfully collect BEVs from a variety of bacteria, such as Gram-negative bacteria (such as *E. coli* Nissle 1917 and *Akkermansia muciniphila*), and Gram-positive bacteria (such as* Lactobacillus rhamnosus GG*). Generally, the bacteria and their debris in fermentation broth are completely removed by low-speed centrifugation (2000 g ~ 1000 g) and 0.22 μm sterile filter. Then, the non-BEVs associated proteins are eliminate by 100 kDa ultrafiltration membrane. Further, the BEVs are isolated and purification by ultracentrifugation (100000 g) and iodixanol gradient centrifugation. The physicochemical properties of collected BEVs can be characterized by transmission electron microscopy (TEM), nanoparticle tracking analysis (NTA), and western blotting (WB), if necessary 57.

## Internalization of BEVs

**Figure 4:**
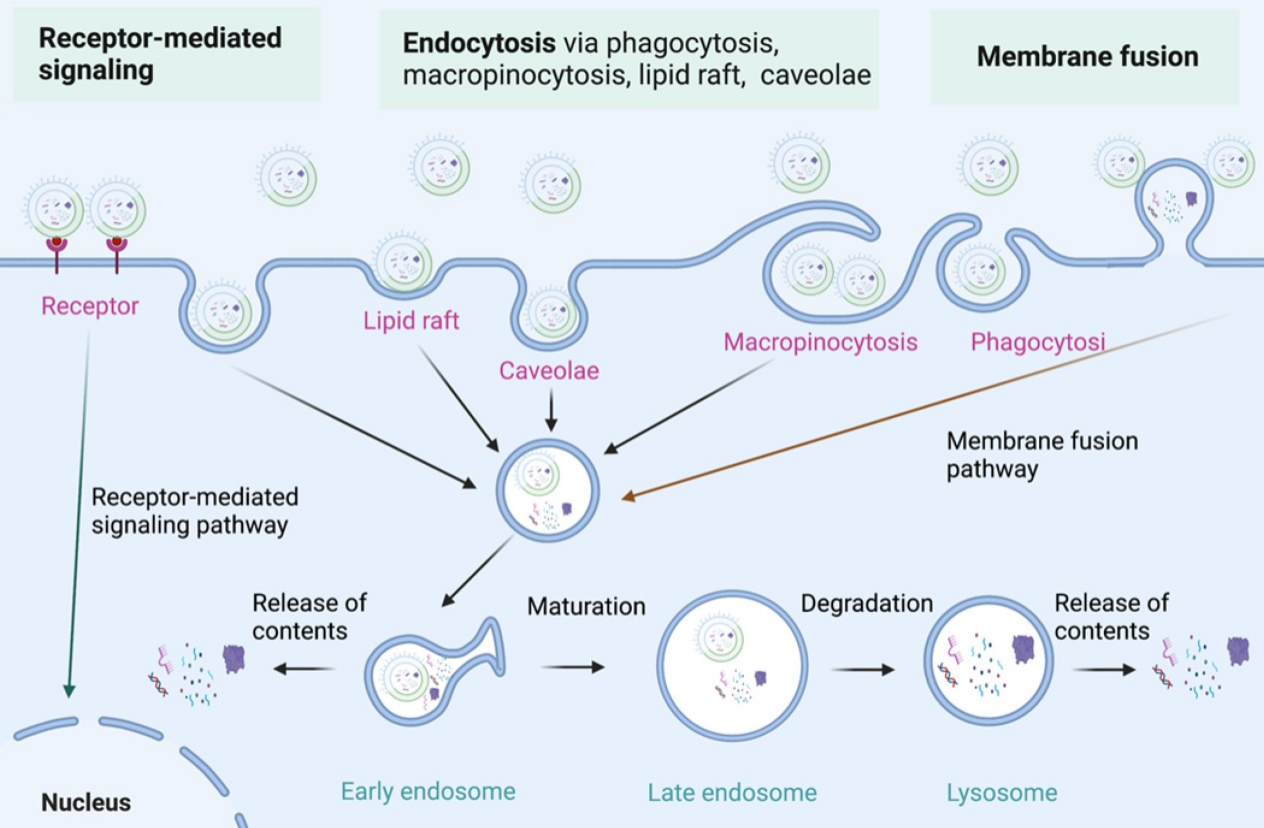
** The internalization of BEVs.** Three major internalization pathways for BEVs internalization have been proposed: 1) Receptor-mediated signaling; 2) Endocytosis via endocytosis via phagocytosis, macropinocytosis, lipid raft, and caveolae; 3) Membrane fusion. Figures were created with https://app.biorender.com/.

As nanoparticles with phospholipid bimolecular membranes, BEVs can be internalized by host cells, which is similar to that of MEVs 58-62. The communication of BEVs and host cells includes the interaction of BEVs with cell receptors, the delivery of agents into cells by BEVs, and the complete entry of BEVs into cells 63, 64. The specific molecular mechanism of BEVs internalization by host cells need further study 65. Currently, three major internalization pathways for BEVs internalization have been proposed (**Figure 4**) 66, 67: 1) Endocytosis; 2) Membrane fusion; 3) Receptor mediated signalling. Endocytosis is the primary mode of BEVs internalization by host cells. Endocytosis and membrane fusion allow BEVs enter into early endosomes, thereby releasing their contents. Moreover, BEVs can also communicate with host cells through toll-like receptors (such as TLR1, TLR2, TLR4, and TLR6)-mediated signaling. The internalization of BEVs initiates a series of responses of host cells, which lays the foundation for the use of BEVs in cancer therapy.

## Sources of BEVs in cancer therapy

Bacteria-based cancer treatment has received many attentions due to the high immunogenicity, which can recruit more immune cells to kill tumor 68-70. However, the application of intact bacteria may introduce safety concerns, such as excessive infection and sepsis 71, 72. Therefore, the BEVs, which have many pathogen-associated molecular patterns (PAMPs), LPS, PG, and bacterial nucleic acids, for instance, have been another superior option for cancer treatment 38, 73, 74.

The PAMPs of BEVs can be engage with host pattern recognition receptors (PRRs) in immune and nonimmune cells to activate immunomodulatory 75. It's worth noting that the immune response elicited by BEVs is mainly dependent on the parental strain and the relationship between parental strain and its host 72, 76. For example, the BEVs derived from pathogenic bacteria, such as *Escherichia coli* (*E. coli*), can mediate the activation of caspase-11 66 or intrinsic apoptosis 77. In contrast, the BEVs produced by symbiotic bacteria, such as *Lactobacillus rhamnosus* GG, *E. coli* Nissle 1917, and *Akkermansia muciniphila* can protect the intestinal epithelium and activate the immune and defense responses 78-80. In addition to these probiotics, many researchers have utilized *msbB* mutant strains as starting strains for subsequent applications 35, 38, 81. The deletion of *msbB* results in under-acylated LPS and exhibits reduced endotoxicity and immunogenicity to human cells 49, 82. Moreover, similar attenuated strains could be obtained by using lysozyme to remove the cell wall 83, 84. Here, we comprehensively summarize the most commonly used bacterial sources for BEVs-based tumor therapy, mainly including pathogenic strains, attenuated strains, and probiotics (Table 2). The summary of sources of BEVs in cancer therapy lays a good foundation for BEVs-based therapy for BSTTs.

## BEVs-based cancer treatment strategies

**Figure 5:**
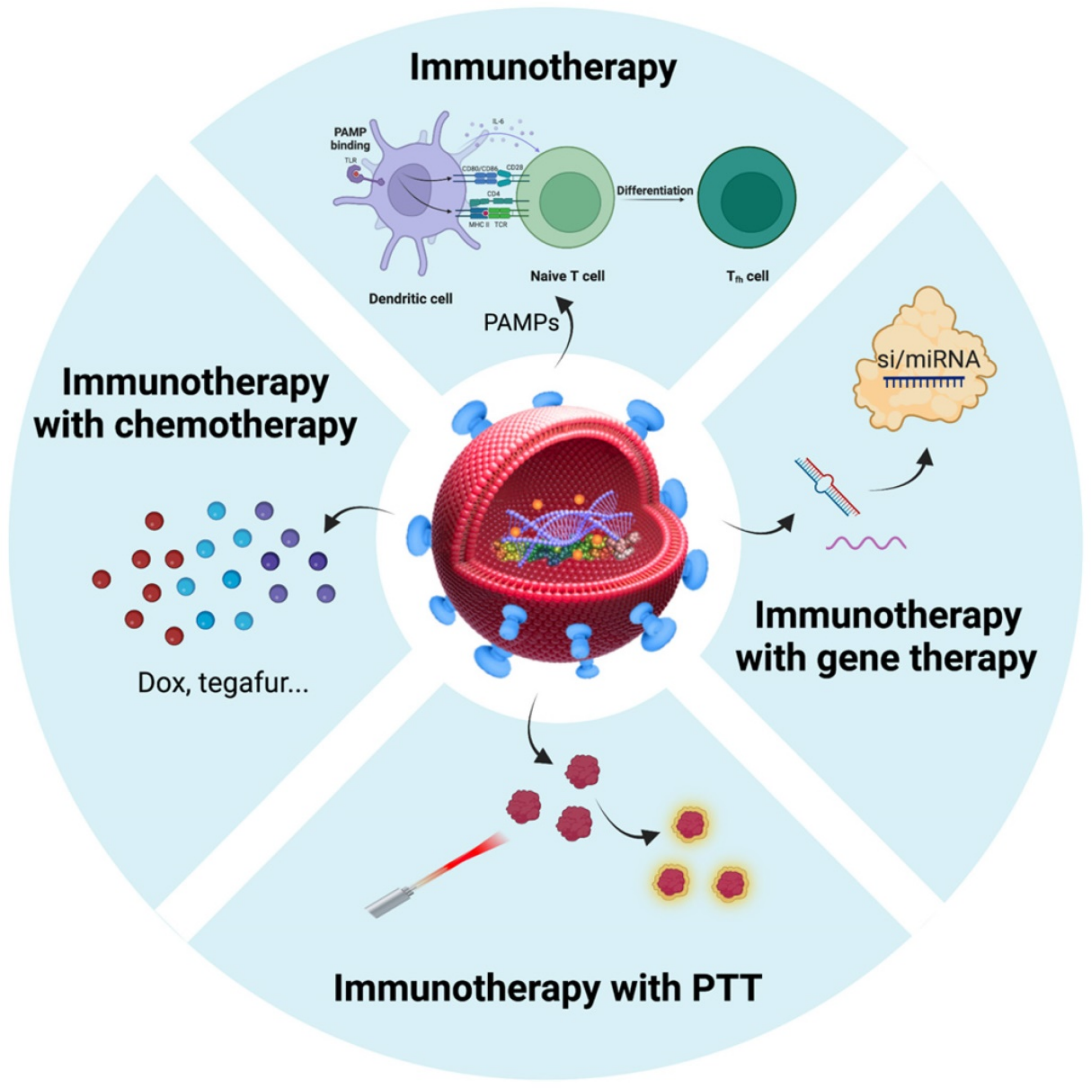
** Summarization of BEVs-based cancer treatment strategies.** Immunotherapy is an important therapy in the field of BEV-based cancer therapy. In addition to immunotherapy, BEVs have been applied to the combination with chemotherapy, gene therapy, and photothermal therapy to amplify antitumor efficacy. Nanostructured BEVs enable efficient lymphatic drainage when injected subcutaneously and enhance localization to solid tumors through passive targeting effect when injected systemically. More importantly, the targeting ability of BEVs can be enhanced by displaying specific proteins on the membrane surface, which can greatly enhance local drug concentration and reduce side effects. Figure was created with https://app.biorender.com/.

After considering the source of BEVs in cancer therapy, we focused on BEVs-based cancer treatment strategies. The properties of BEVs of immunogenicity, cell-free system, safety, and nanoscale structure endow them with the potential to treat cancer 103. Nanostructured BEVs enable efficient lymphatic drainage when injected subcutaneously and enhance localization to solid tumors through passive targeting effect when injected systemically 104. Importantly, another major advantage of BEVs in cancer applications is the ease of genetic engineering editing that can confer various functions on BEVs 39, 40, 105. Therefore, in addition to immunotherapy, BEVs have been applied to the combination with other types of therapies (such as chemotherapy, gene therapy, and photothermal therapy) to synergistically amplify antitumor efficacy (Table 2). Here, we summarized the BEVs-based cancer treatment strategies (**Figure 5**), which may provide constructive guidance for the treatment of BSTTs.

## Immunotherapy

Immunotherapy, an important therapy in the field of BEV-based cancer therapy, has revolutionized the clinical treatment of cancer 106. The breakthroughs of immunotherapy have shown great potentials over the last decade 107. As mentioned above, the PAMPs can coffer excellent intrinsic immunomodulatory properties on BEVs, which are often used as pathogen mimicking adjuvants 47, 108. BEVs-based immunotherapeutic, such as Bexsero and MeNZB, have been approved for clinical treatment of meningococcal group B infections 47. It has been reported that intravenous injection of attenuated* E. coli* derived BEVs elicited a strong and long-term IFN-γ and T cell mediated antitumor immune response, which could completely eradicate established tumors without significant side effects 81. However, the IFN-γ could upregulate the expression of immune checkpoint programmed death 1 ligand 1(PD-L1), which may induce the dysfunction and apoptosis of T cell by interacting with programmed death 1 (PD1) on its surface, and hence, limit the effectiveness of immunotherapy 109, 110. In turn, inhibiting the interaction of PD-L1 and PD1 could enhance the immune response to the cancer cells. Recognition of tumor antigens by the immune system has been shown to be critical to the success of therapy 111.

**Figure 6:**
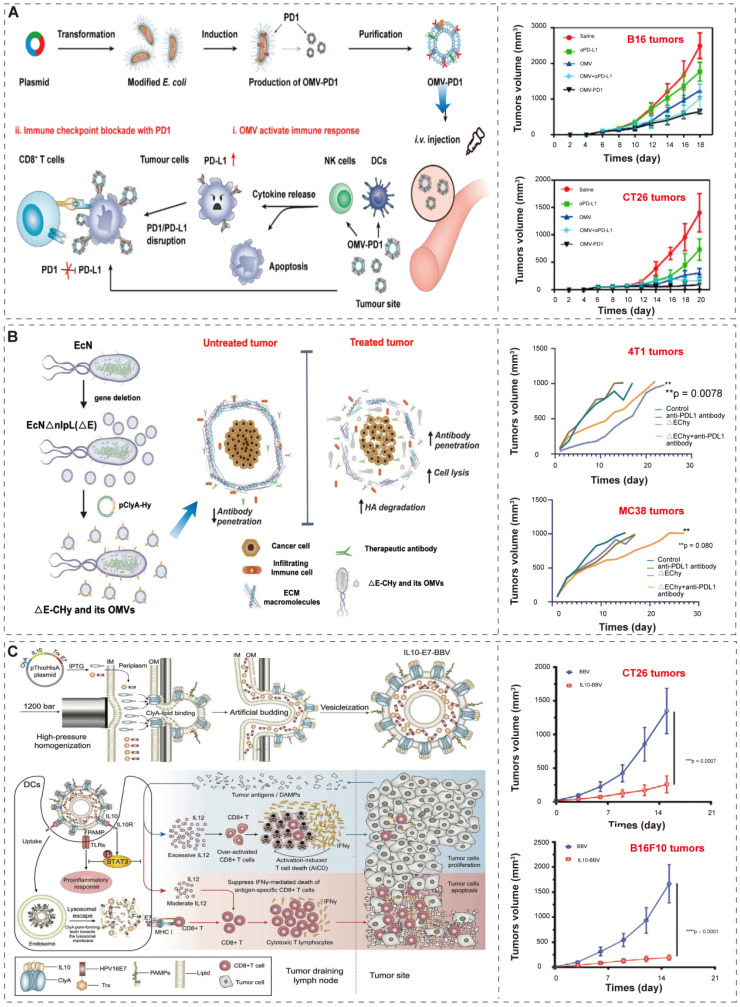
** BEVs-based immunotherapy. (A)** Schematic illustration of the antitumor mechanism and characterization of BEVs-PD1 for cancer immunotherapy. Adapted with permission from 74, copyright 2020, American Chemical Society. **(B)** Schematic illustration of the construction of EcN into ΔE-CHy and its application. Adapted with permission from 87, copyright 2022, Wiley-VCH GmbH. EcN, *E. coli* Nissle 1917; ΔE, EcN with the deletion of* nlpI*; CHy, ClyA-Hy. **(C)** Schematic illustration of the construction and the mechanism of multifunctional modified biomimetic BEVs. Adapted with permission from 94, copyright 2021, Wiley-VCH GmbH. It is worth noting that the author uses OMV to represent the extracellular vesicles produced by bacteria, which is not exactly the same as the OMVs described in “2.1 Biogenesis, composition, and classification of BEVs*”*. In order to maintain the consistency of the article, we use BEVs to represent the extracellular vesicles produced by bacteria, and the explanation will not be repeated later.

Recently, Li et al. 74 and Cheng et al. 86 developed an efficient “Plug and display” system for displaying exogenous proteins on BEVs. They applied ClyA, a secreted, pore-forming protein 112, 113, to promote the localization of exogenous proteins (such as luciferase, Luc) or antigen (such as PD1) on the outer membrane of bacteria and their secreted BEVs. The bioengineered BEVs-PD1 retain the ability of immune activation. Moreover, the co-incubation of BMVs-PD1 and bone marrow dendritic cells (BMDCs) show great biocompatibility. Further, the BEVs-PD1 can promote the combination of PD-L1 on the surface of tumor cells and protect T cells from the PD1/PD-L1 immunosuppressive axis (**Figure 6A**). In conclusion, the “Plug and display” system based on BEVs offer a broad prospect for tumor immunotherapy.

Furthermore, since BEVs have the excellent intrinsic immunomodulatory properties, different synthetic BEVs have also been developed 84, 94. Considering that desmoplastic solid tumors are characterized by the accumulation of hyaluronic acid (HA), which prevents the infiltration of immune cells 114, Thomas et al.^87^ used “Plug and display” system to display hyaluronidase (Hy) instead of antigen on probiotic *E. coli* Nissle 1917 derived BEVs (**Figure 6B**). BEVs-Hy can reconstruct the tumor microenvironment, enabling immune checkpoint antibodies and tyrosine kinase inhibitors work together to exert immunotherapy effects (**Figure 6B**).

Moreover, Hua et al. 94 utilized high-pressure homogenization to produce biomimetic BEVs with immunomodulator IL-10 (**Figure 6C**). These biomimetic BEVs platform have a promising potential in cancer immunotherapy (**Figure 6C**). Similarly, Park et al. 84 applied high lysozyme and high pH treatment of bacteria to generate synthetic BEVs, which contained few cytosolic ingredients and nucleic acid (such as RNA and DNA). Compared to conventional BEVs, these BEVs were safer and did not induce systemic pro-inflammatory factors *in vivo*, and successfully induce tumor regression in melanoma mice.

## The combination of immunotherapy and chemotherapy

Broad-spectrum antitumor drugs, such as doxorubicin (DOX) and tegafur, are often used to treat tumors 115. However, chemotherapy drugs have no specific targeting and have obvious side effects, which often lead to poor therapeutic effects 40. Therefore, there is a need to develop safer and more effective drug delivery systems to alleviate toxicity and enhance the antitumor effect of drugs.

**Figure 7:**
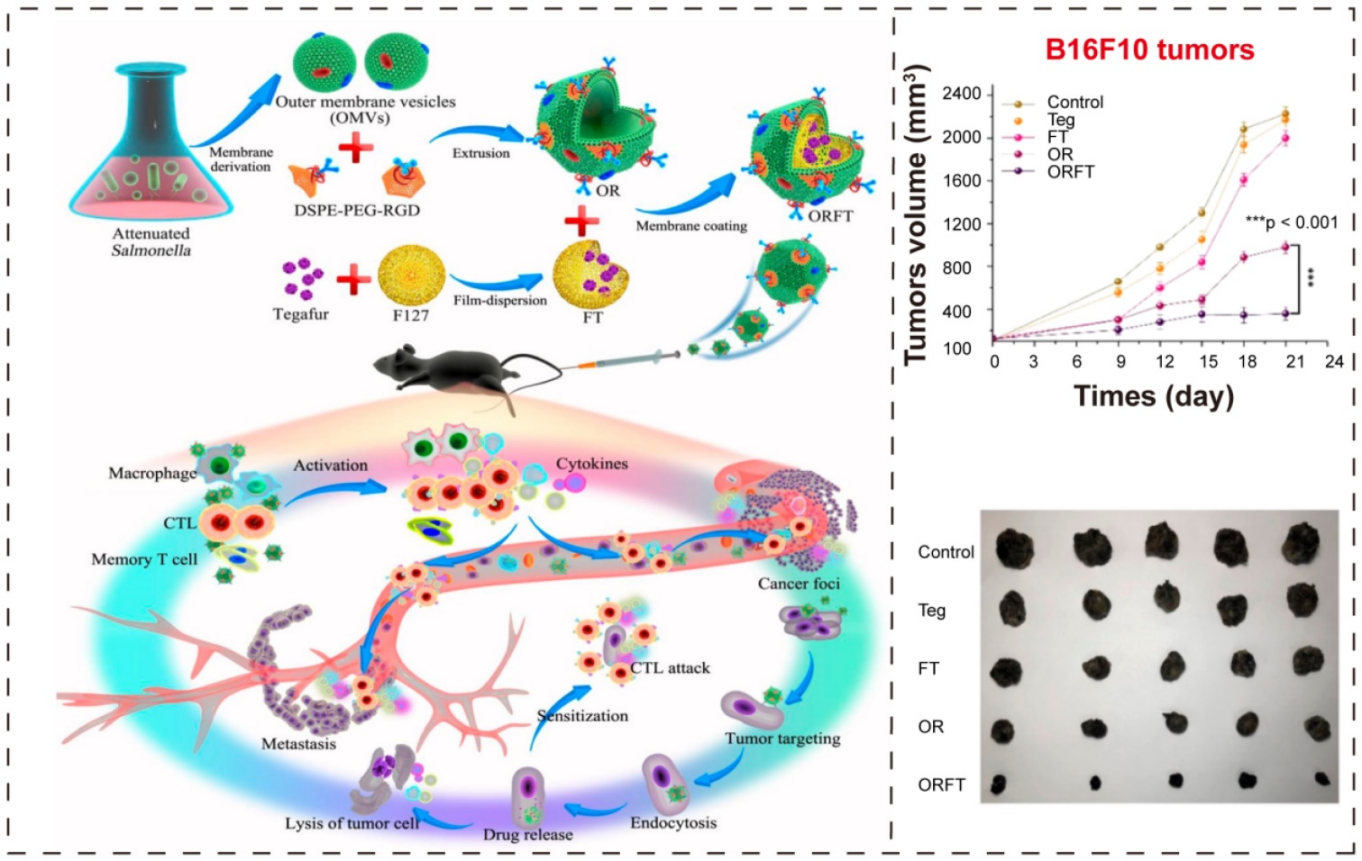
**BEVs-based immunotherapy and chemotherapy. (A)** Schematic illustration of the construction of bioengineered BEVs-coated polymeric micelles and the effect of combination of immunotherapy and chemotherapy. Adapted with permission from 97, copyright 2020, American Chemical Society.

In a recent report, Chen et al. 97 proposed an ingenious combinational strategy where attenuated *Salmonella* derived BEVs coated polymeric nanomedicine to improve the immunotherapeutic efficacy (**Figure 7A**). Here, they exploited BEVs as initiator of antitumor immune response, and utilized RGD (Arg-Gly-Asp) 116 as targeting ligand to enhance the tumor-targeting ability, and used tegafur, a prodrug of fluorouracil 117, 118, as chemotherapeutic drug to amplify the immunotherapeutic potential of BEVs. The BEVs-coated hybrid nanoparticles could simultaneously show chemotherapeutic and immunological efficacy, sensitizing melanoma cells to cytotoxic T lymphocytes (CTLs), further achieving noticeable inhibition of cancer metastasis.

Kuerban et al. 96 obtained engineered BEVs derived from attenuated *K. pneumonia*, and then loaded with DOX, one of the most widely used antineoplastic drugs 119, by incubated at 37 °C for 4 h. In addition to the appropriate immunogenicity, BEVs-DOX also increased the expression of cleavage caspase-3, cleavage PARP, and F4/80 protein, thereby achieving the purpose of inhibiting tumor growth. Importantly, BEVs-DOX had a better biosafety than free DOX and no side effects of toxicity to major organs. In general, BEVs can not only serve as efficient drug delivery vehicles for chemotherapeutic agents, but also introduce appropriate immune responses.

## The combination of immunotherapy and gene therapy

Gene therapy, a method to compensate or correct mutant genes in tumor cells by delivering gene regulators, such as miRNA, siRNA etc., has shown significant therapeutic effects and great safety record and has proven to be an extremely promising approach to cancer treatment 120-122. Generally, miRNAs or siRNAs are well known for their poor stability, short half-life, and poor penetration capacity 123, 124. Therefore, the efficient delivery for customized gene regulator is still a challenge. Currently, lipid nanoparticles are the major carriers for RNA delivery in clinical practice 125, 126. However, in tumor treatment, these lipid-based nanoparticles often require the addition of immune adjuvants to activate adaptive immune, which complicates the material preparation process 127, 128. As mentioned above, the BEVs have the excellent properties of intrinsic immunoregulation and drug delivery. Therefore, the BEVs are the ideal nanocarriers for the combination of immunotherapy with gene therapy.

**Figure 8:**
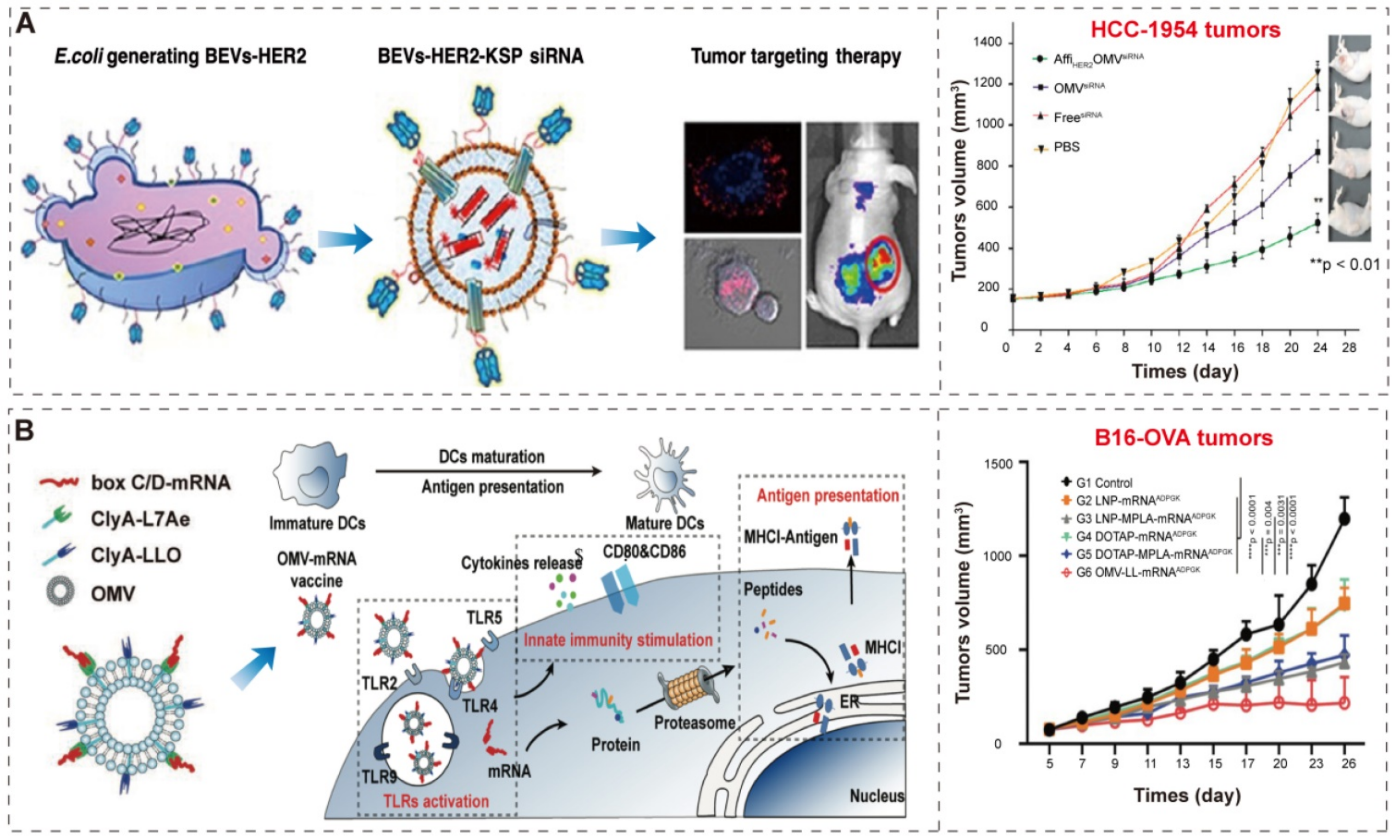
** BEVs-based immunotherapy and gene therapy. (A)** Schematic representation of the BEVs-based siRNA delivery system, which displays HER2 affibody in the outer membrane to specifically target tumors. Adapted with permission from 38, copyright 2014, American Chemical Society. **(B)** Schematic representation of the BEV-based mRNA delivery system and innate immunity activation and antigen presentation. Adapted with permission from 98, copyright 2022, Wiley-VCH GmbH.

Gujrati et al. 38 constructed bioengineered BEVs to deliver kinesin spindle protein (KSP) siRNA (**Figure 8A**). KSP is abundantly overexpressed in tumor tissues to regulate cell cycle progression; the silencing of KSP mRNA expression results in the arrest of cell cycle and the induction of apoptosis 129, 130. The bioengineered BEVs, derived from attenuated *E. coli* W3110△*msbB*, display human epidermal growth factor receptor 2 (HER2) affibody in the outer membrane to specifically target tumors through the “Plug and display” system (ClyA-Luc). Then, KSP siRNA was loaded into BEVs-HER2 by conventional electroporation method 131. Finally, the bioengineered BEVs-HER2-KSP siRNA induced obvious tumor growth regression without nonspecific side effects.

In a recent study, Li et al. 98 developed BEVs-based box C/D mRNA delivery platform by ClyA-L7Ae (L7Ae, an archaeal RNA-binding protein 132, 133) and CylA-LLO (LLO, a lysosomal escape protein 134). The BEVs-L7Ae-LLO can combine box C/D mRNA through L7Ae binding, resulting in BEVs-L7Ae-LLO-mRNA. Then, BEVs-L7Ae-LLO-mRNA deliver the mRNA into dendritic cells for the purpose of antigen presentation and innate immune stimulation (**Figure 8B**). The mRNA vaccines can encode one or more tumor-specific antigens (TSA), and undergo protein translation and antigen processing in cells, and bind to major histocompatibility antigen complex I (MHCI) in antigen-presenting cells, and finally presented to T cells to induce a strong tumor-specific T cell response to kill tumor cells 135, 136. In addition, the innate immunity by BEVs enhanced the activation of antigen-specific T cells, which in turn significantly inhibited the tumor progression. The BEVs based mRNA delivery indicates the great potential of BEVs as a vehicle for combined immunotherapy and gene therapy in the treatment of tumors.

## The combination of immunotherapy and photothermal therapy

Photothermal therapy (PTT) is one of the latest tumor treatment options that applies photothermal transduction agents (PTAs) with photothermal conversion efficiency and converts light energy into heat energy under the near-infrared (NIR) laser to kill cancer cells 137-139. The tumor-specific cytotoxic T cells are activated by tumor antigens released by heat-damaged tumor cells 140. Generally, compared with traditional surgery, chemotherapy, and gene therapy, PTT is a more low-toxic and minimally invasive tumor-targeted therapy. In the past three years, there have been numerous success cases in the treatment of tumors through the combination of photothermal therapy and BEVs-based immunotherapy 35, 99-102.

**Figure 9:**
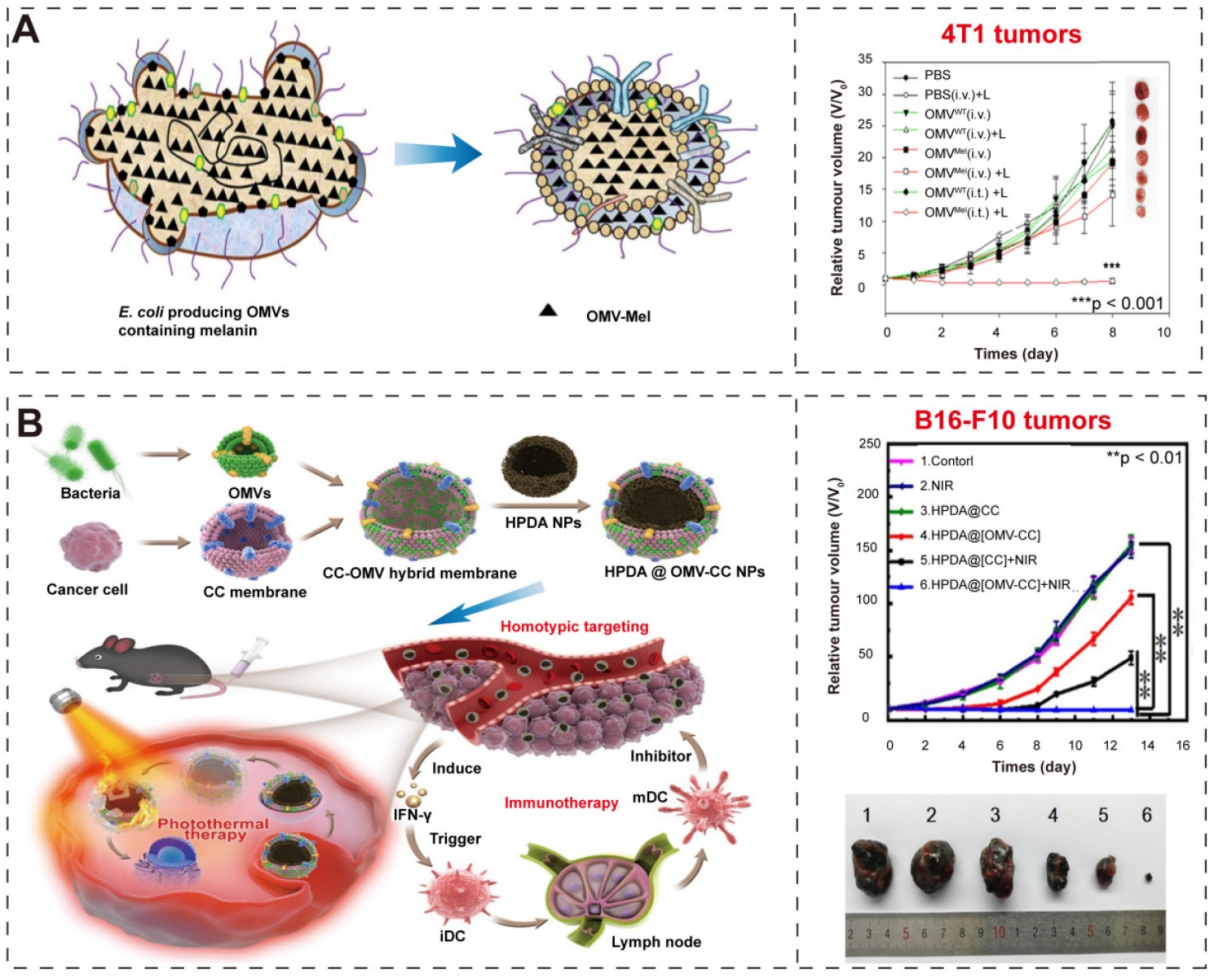
** BEVs-based immunotherapy and photothermal therapy. (A-B)** Schematic illustration of BEV-Mel production. Adapted with permission from 35, copyright 2019, Springer Nature. **(C-D)** Schematic illustration of the construction of HPDA@BEV-CC NPs and their antitumor immune responses after PTT. Adapted with permission from 99, copyright 2020, American Chemical Society.

Gujrati et al. 35 overexpressed biopolymer-melanin (Mel) in the attenuated* E. coli* W3110△*msbB* and obtained the bioengineered BEVs-Mel, which could produce appropriate optoacoustic signals for imaging applications in cancer photothermal therapy (**Figure 9A-B**). Although the exact mechanism for Mel encapsulation is not known, the entire process is a natural event that does not require any complex synthetic skills, and BEVs-Mel can be obtained in large quantities through cost-effectively, well-established large-scale bacterial culture 32-34. Importantly, the BEVs-Mel platform did not induce chronic systemic toxicity and side effects despite repeated injections into mouse tumor models. Except for Mel, the hemoglobin can also be used in PTT cancer treatment 141. Zhuang et al. 102 proposed a new strategy, which applying BEVs to induce extravasation of red blood cells (RBCs) in tumors, thereby accumulating hemoglobin (one of PTAs). Compared with previous BEVs-based cancer treatments 38, 81 that required high-dose injection, the combination of BEVs with PTT enables better efficacy with only low-dose injection, which significantly reduces side effects and toxicity concerns caused by BEVs injection.

Given that the efficiency of tumor antigen recognition and operation by the immune system is also important for successful immune activation after PTT 142, 143. Therefore, Li et al. 100 presented the BEVs-based multifunctional NPs, which have the ability of antigen capture and immunomodulation. The native BEVs was first modified with maleimide (Mal), which can bind proteins/antigens via thioether bonds. Then, they loaded them with 1-methyl-tryptophan (1-MT, an inhibitor of indoleamine 2, 3-dioxygenase 144), producing the 1-MT@BEV-Mal NPs. The cancer cells were incubated with 20 μg/mL indocyanine green (ICG, one of PTAs) for PTT. These BEVs-based NPs possesses antigen capture and immunomodulation to promote immune-mediated tumor clearance after PTT.

In addition, the hybrid membrane nanoplatforms provide a promising biomimetic strategy in cancer treatment. Wang et al. 99 constructed hybrid nanoparticles (NPs) by using attenuated BEVs and B16-F10 cancer cells (CCs) membrane and then coated them onto hollow polydopamine (HPDA, one of PTAs 145) (**Figure 9C-D**). They used these nanoparticles to target melanoma by combining immunotherapy with BEVs and HPDA-mediated photothermal therapy, and finally successfully eradicated melanoma without significant side effects. Chen et al. 101 also designed a hybrid membrane eukaryotic-prokaryotic vesicles (EPVs) with tumor-specific antigenic by fusing eukaryotic cancer cell membrane vesicles (CCMVs) and prokaryotic BEVs. Subsequently, they conferred these EPVs with PTT module by coating with poly (lactic-co-glycolic acid)-ICG nanoparticles 146, 147 (PLGA-ICG NPs, PI), resulting in PI@EPV NPs. The PI@EPV NPs obtained two immunological functions from their parent membranes and the PTT module, which could enhance the immunotherapeutic effects and destroy the solid tumor.

## Potential role of BEVs in BSTTs

Osteosarcoma, chondrosarcoma, and Ewing sarcoma are the three most common primary malignant bone tumors. The pathological types of soft tissue tumors are complex, the most common of which are undifferentiated pleomorphic sarcoma, liposarcoma and leiomyosarcoma 148. Conventional BSTTs treatment approaches include radiation therapy, surgery, and chemotherapy 149. With the understanding of the pathogenesis and progression of BSTTs, disrupting the receptors of vascular endothelial growth factor (VEGF) and platelet-derived growth factor (PGDF) that control angiogenesis, hindering the formation of the mitotic spindle, disturbing the late S-phase and G2 phase of the cell cycle, and inhibiting the association of DNA-binding proteins are considered to be essential for the management of these tumors 148. Therefore, many targeted drugs such as Pazopanib 150, Sorafenib 151, Eribulin 152, and Trabectedin 153 have been developed for the treatment of the diseases. In addition, recent basic and clinical studies have confirmed the relationship between immune checkpoints and malignant tumor progression, as well as the efficacy of immune checkpoint inhibitors on various malignant tumors 154-156. However, these antitumor drugs have not achieved satisfactory efficacy due to the rarity and diversity of BSTTs.

In recent years, it has been confirmed that the metabolites derived from disturbed gut microbiota affects the progression of BSTTs 11, 12. For example, compared with healthy subjects, the gut microbiota of multiple myeloma (MM, also known as plasma cell myeloma, an incurable tumor that accumulate in the bone marrow 157) patients is rich in opportunistic nitrogen-cycling bacteria and produces more available L-glutamine, which accelerates MM progress 158. In conclusion, there is a strong link between commensal bacteria and these tumors. Therefore, it is possible to apply fecal microbiota transplantation (FMT) to the treatment of BSTTs 159. FMT is an emerging therapeutic approach that affects the course of a variety of chronic diseases, including metabolic syndrome, autoimmune, and cancer 160, 161. However, manipulating the gut microbiome also carries certain risks 162. BEVs are cell-free nanocarriers that carry a variety of key bioactive contents and PAMPs derived from parental strain. From a therapeutic perspective, direct systemic administration of BEVs derived from symbiotic bacteria in healthy hosts to tumor-bearing hosts may be a better alternative than of FMT 159.

**Figure 10:**
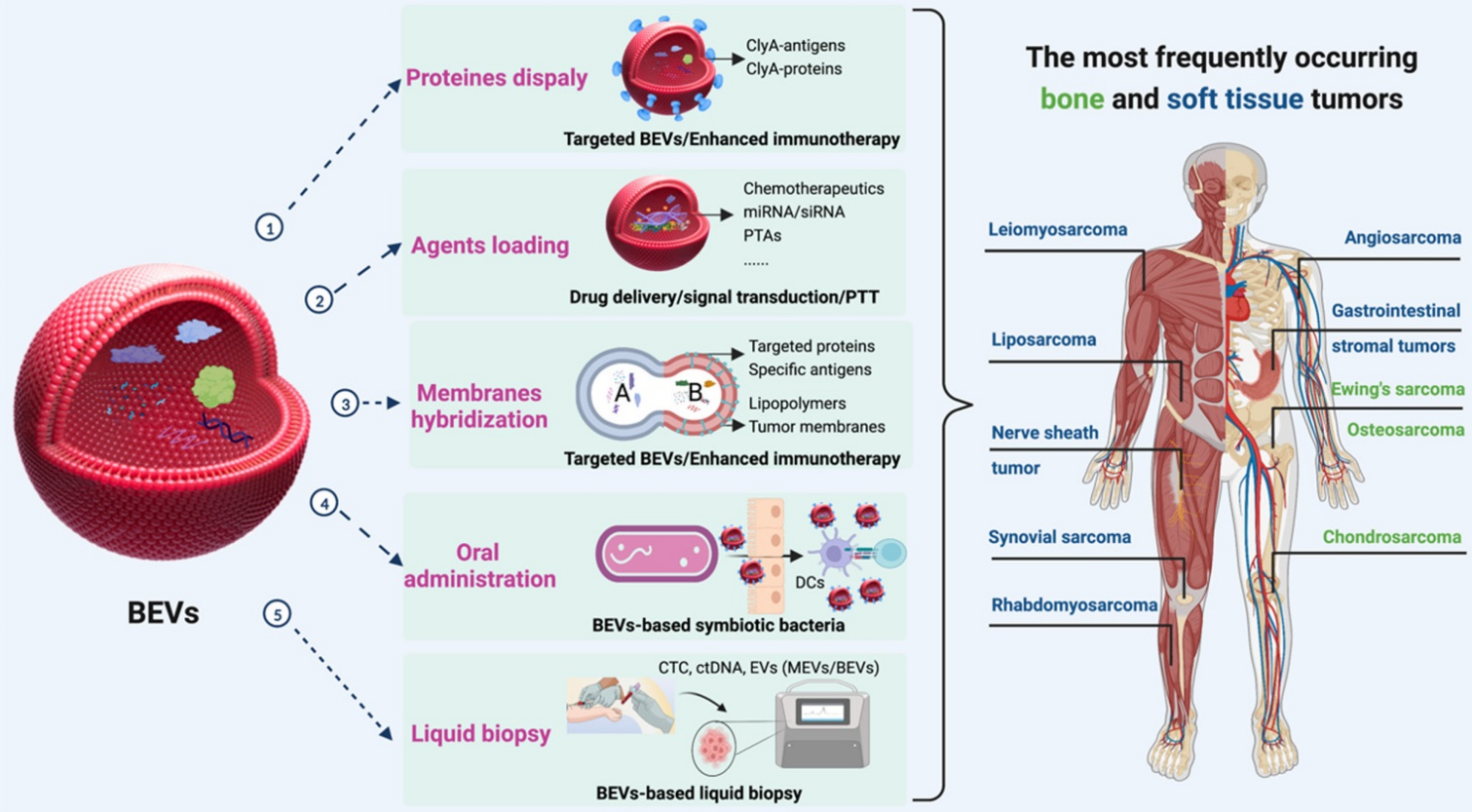
** The potential role of BEVs in BSTTs.** Displaying proteins or antigens on the membranes, loading therapeutic agents (such as chemotherapeutics, miRNA, siRNA, and PTAs), and hybridizing other functionalized biological membranes (such as lipopolymers and tumor membranes) can be used to enrich the therapeutic and targeting functions of BEVs for BSTTs. Moreover, oral administration of BEVs or BEVs-based symbiotic bacteria will be one of the promising directions of BEVs-based cancer therapy. In addition, another important application area of BEVs is the diagnostic biomarker in tumor liquid biopsies. Figure was created with https://app.biorender.com/.

Furthermore, the combination of immunotherapy with other types of therapy is a powerful strategy for enhancing antitumor responses 163, 164. Three schemes can be used to enrich the functions of BEVs for BSTTs (**Figure 10**). The first approach is to display proteins and antigens on the membranes to enhance the immunotherapy or targeted ability through the “Plug and display” system. The second solution is to apply the drug loading capacity of BEVs to achieve synergistic therapy with other therapeutic approaches by delivering therapeutic agents such as Doxorubicin, Pazopanib, miRNA, and PTAs to BSTTs cells. The third method is to hybridize other functionalized biological membranes such as lipopolymers and tumor membranes with BEVs to obtain new functions.

Currently, BEVs-based cancer therapies are administered either subcutaneous injection or intramuscular injection, where the immune activation is controlled by limited draining lymph nodes, and thus resulting in insufficient immunogenicity 165. In addition to enhancing tumor antigen display and the combination of multiple therapies, oral administration of BEVs or BEVs-based symbiotic bacteria will be the future direction of BEVs-based cancer therapy (**Figure 10**). Lately, Yue et al. 95 proposed a BEVs-based oral tumor vaccine for specific immune activation. Compared with subcutaneous or intramuscular injection, oral administration may offer better safety, better patient compliance, and lower healthcare cost to BSTTs patients 166.

In addition, another important application area of BEVs is the diagnostic biomarker in tumor liquid biopsies (**Figure 10**) 167-169. Tumor EVs are gradually developing as a third liquid biopsy marker besides circulating tumor cell (CTC) and circulating tumor DNA (ctDNA) 170, 171. In recent years, studies have shown that microbial colonization also exists in tumors 172, and these bacteria can also affect host behavior such as metastasis 173. With the advancement of omics, important proteins, nucleic acids, and lipids, potential liquid biopsy markers, can be identified in BEVs isolated from BSTTs.

## Major advantages and challenges of BEVs

BEVs have been regarded as a source of revolutionary nanotechnology therapeutics, which have a number of advantages that make them attractive for cancer therapy 30. Compared with MEVs, the unique advantages of BEVs in cancer therapy are intrinsic immunomodulatory properties, ease of industrialization, and ease of customization. Although numerous reports have demonstrated that the obvious therapeutic effects of BEVs in cancer treatment, there is still various challenges to be conducted to move them from the laboratory to clinical applications. Here, we summarize the major advantages and challenges of BEVs in cancer therapy (Table 3).

## Advantages

### Intrinsic immunomodulatory properties

Compared with other therapeutical nanoparticles, such as exosomes (or MEVs) 174-176, lipidosome 177, and metal nanoparticles 178, 179, one of the unique advantages of applying BEVs into cancer therapy is that the intrinsic immunomodulatory properties elicited by PAMPs. Therefore, in addition to immunotherapy, BEVs-based therapies can synergize with a variety of other therapies such as chemotherapy, gene therapy, and photothermal therapy to enhance antitumor effects.

### Ease of industrialization

Despite the considerable success of synthetic nanomaterials in the treatment of diseases such as cancer in preclinical trials, only a few synthetic drugs have entered clinical trials 180, 181. The factors that limiting the clinical application of most synthetic nanomaterials include challenges involving material-related toxicity, low biocompatibility, and high cost of large-scale production 182, 183. Bacteria-based fermentation has always been an economical, scalable, and environmentally friendly technology 32-34. The rapid proliferation and large-scale fermentation processes of bacteria provide a scalable and powerful platform to produce a large number of cell-free BEVs to meet commercial and clinical needs.

### Ease of customization

The rapid development of synthetic biology brings infinite possibilities for engineering editing of bacteria 184, 185. The therapeutic components of interest can be easily loaded inside or outside the BEVs by manipulating their parent bacteria. Importantly, the biogenesis mechanism of BEVs such as explosive cell lysis for Gram-negative bacteria and bubbling cell death for Gram-positive bacteria make customization of BEVs possible. Moreover, in addition to customized modification in bacteria, we can also perform personalized modification after isolation of BEVs through engineering techniques such as membrane fusion 186, membrane coating 187, covalent reactions 188, and noncovalent reactions 189.

### Higher safety

Bacteria, especially the attenuated bacteria, based cancer immunotherapies have attracted much attention due to their unique ability to trigger host antitumor immunity 69, 190. Although the virulence and the risk of septic shock have been reduced, the safety of attenuated bacteria still needs to be improved to meet the requirements of clinical application. BEVs, cell-free and non-replicable nanoparticles 191, are considered to be safer than parental strains. Importantly, BEVs contain most of the immunogenic membrane-associated components of their parental strains, which can activate and modulate the immune response even in small injections, thereby improving their safety *in vivo* studies 81, 192.

## Challenges

### Lack of standardization

A definite standard for BEVs production, isolation, and characterization has still not been achieved, which seriously hinders the commercial and clinical application of BEVs. In the process of fermentation, especially at large-scale, changes in culture medium (such as carbon source, nitrogen source, trace elements), pH, temperature, and regulation modes etc. will affect the metabolic process of bacteria, thereby affecting the production, size, and composition of BEVs 65. Actually, the expression of some heterologous proteins, such as PD1, needs to be induced under low temperature conditions (such as 18°C 74), but this condition may not be suitable for the mass production of BEVs. Moreover, the methods of BEVs isolation remains controversial. Although there are a variety of widely recognized purification schemes 39, most of them require repeated and time-consuming ultracentrifugation and ultrafiltration. The existing commercial purification kits are faced with high price and low purity. Furthermore, natural MEVs have abundant and clear specific biomarkers in the membrane, such as CD81, CD63, CD9, and TSG101 20. However, BEVs are lack of definite individual markers, which is not conducive to the characterization of BEVs 193. In conclusion, it is urgent to determine a definite standard for BEVs production, isolation, and characterization.

### Potential off-target effects

In addition to being an activator of immune response, BEVs can also be used as drug delivery systems to deliver chemotherapeutic drugs and gene disruptors in BEVs-based cancer therapy. These exogenous cargoes loaded in BEVs may interfere or interact with endogenous cargoes, that is off-target effects 58. The heterogeneity, including size, content, functional and source heterogeneity, of BEVs will become a prominent obstacle if off-target effects are excessive, especially in cancer gene therapy 20, 58. For example, single miRNA may not completely cure the disease when the off-target effects exceed the therapeutic goal, and thus, a family of miRNAs may be needed to ensure that upstream and downstream targets are modulated 58. Therefore, reducing the off-target effects is of great concern when constructing therapeutic BEVs.

### Potential biosafety

It must be pointed out that “Potential biosafety” in the challenges does not conflict with “Higher safety*”* in the advantages. Although many BEVs-based cancer therapies have confirmed that appropriate injection of BEVs does not cause any significant side effects 83-91, we still need to evaluate the safest bacteria source for obtaining therapeutic BEVs that do not deliver unwanted toxic substances and cause incalculable problems 194. In addition, *in vivo* experiments are still needed to clarify the distribution, dose, and clearance rate of BEVs to comprehensively evaluate the safety of BEVs 95.

### Ambiguous contents

The intrinsic composition of BEVs is plentiful, for example, natural *E. coli* DH5α derived BEVs have been identified 141 proteins by global proteomic profiling 195, which may contain the unwanted toxic substances or off-target effects substances. Therefore, Park et al. 84 obtained synthetic BEVs with few cytosolic ingredients and nucleic acid by applying high lysozyme and high pH treatment of bacteria. Although many BEVs-based cancer therapeutic strategies have successfully induced significant regression of tumor growth without significant side effects, the content of these BEVs remains ambiguous 69, 190. Fortunately, with the development of high-throughput sequencing and omics, a clear and complete map of contents of BEVs will be presented to us, so that BEVs-based cancer treatment can be more refined.

## Conclusions and perspectives

In this review, we outlined the biogenesis, composition, isolation, classification, and internalization of BEVs. Gram-negative bacteria derived BEVs are generated by explosive cell lysis and blebbing of the outer membrane, while the biogenesis mechanism of Gram-positive bacteria is bubbling cell death. According to the different biogenesis mechanisms, BEVs can be divided into four categories, OIMVs, EOMVs, OMVs, and CMVs. For composition, OMVs mainly contain outer membrane proteins, and other BEVs contain a large number of proteins, nucleic acids, metabolites, etc. Moreover, an efficient BEVs isolation strategy based on ultracentrifugation and density gradient centrifugation is provided. When considering the interaction of BEVs with cells, three main internalization mechanisms, including endocytosis, receptor mediated signalling, and membrane fusion, have been identified.

We then comprehensively summarize the sources of BEVs in cancer therapy. The sources of BEVs used for cancer treatment are widely distributed, ranging from pathogenic bacteria (such as *E. coli* Rosetta, *E. coli* DH5α, and *E. coli* TOP10) to attenuated bacteria (*E. coli* W3110△*msbB*), and to probiotics (such as *E. coli Nissle 1917*, *Akkermansia muciniphila*, and* Lactobacillus rhamnosus GG*). Subsequently, the BEVs-related cancer treatment strategies are comprehensively summarized. In addition to immunotherapy, BEVs have been applied to the combination with chemotherapy, gene therapy, and photothermal therapy to synergistically amplify antitumor efficacy. Based on it, we propose the potential role of BEVs in BSTTs. After a comprehensive discussion in the above sections, we highlight the major advantages and challenges of BEV in cancer therapy. The BEVs of cancer treatment display various characteristics such as intrinsic immunomodulatory properties, ease of industrialization, ease of customization, and higher safety. However, there are still challenges, including lack of standardization, potential off-target effects, potential biosafety, and ambiguous contents, to move BEVs from lab to clinic.

In the past research, our team has accumulated a lot of basic and clinical experiences in bone and soft tissue diseases 196-199. Although the application of BEVs in BSTTs is not as prosperous as MEVs 200, as the relationship between the microbiome and human health becomes clearer, the unique properties of BEVs will make them become another promising approach for these tumors. Furthermore, with the advancement of synthetic biology and molecular biology technology, an increasing number of research have focused on BEVs-based subjects, including BEVs-mediated inflammatory responses, BEVs-based adjuvant, vaccine, and antitumor applications. Moreover, the topic of “nonmammalian EVs, especially BEVs” is considered to be one of the hottest directions 191. Importantly, engineered BEVs will further enhance the efficacy of tumors therapy by increasing the local concentration of the therapeutic agent and minimizing side effects. In conclusion, technical advances and clinical regulatory approvals will further drive the development of BEVs-based cancer therpay including BSTTs. Despite the constant challenges, significant progresses of BEVs in cancer therapy have been made in recent years. Continued study on BEVs-based BSTTs will undoubtedly lead to more innovative solutions to current challenges and achieve clinical applications.

## Figures and Tables

**Table 1:** The major advantages and disadvantages of different BEVs isolation methods

Methods	Advantages	Disadvantages	References
Ultracentrifugation	Simple process; Great homogeneity; Lower costs.	Limited efficiency; Limited purity; Time-consuming.	39
Ultrafiltration	Simple process; Great homogeneity; High recovery rate.	Limited efficiency; Limited purity; Product loss.	55
Precipitation	Simple process; Lower costs; Suitable for large sample	Limited efficiency; Limited purity; Time-consuming.	56
Affinity isolation	High purity	High cost; Low availability.	56
Size exclusion chromatography	High purity; High biological properties	Time-consuming; Not suitable for large sample.	55
Density gradient centrifugation	High purity	High cost; Time-consuming.	40

**Table 2:** Summary of the sources of BEVs and different therapies in cancer therapy

Therapeutic strategy	Cancer cells	BEVs source	References
Immunotherapy	B16-F10 and CT26	Attenuated *E. coli* W3110△*msbB*	74
Immunotherapy	B16-BL6, CT26, 4T1, and MC38	Attenuated* E. coli* W3110△*msbB*	81
Immunotherapy	B16-F10 and B16-OVA	*E. coli* Rosetta (DE3)	85, 86
Immunotherapy	4T1, Panc1, and MC38	Probiotics *E. coli Nissle 1917*△*nlpI^a^*	87
Immunotherapy	MDA-MB-468	Attenuated* E. coli* W3110△*msbB*△*pagP^b^*	88
Immunotherapy	B16-F10	Attenuated* E. coli DH5α*	84
Immunotherapy	TC-1	Attenuated* E. coli DH5α*	89
Immunotherapy	B16-F10, 4T1, EMT6, and CT26	Attenuated* E. coli DH5α*	83
Immunotherapy	HTC116, MCF-7, and HepG2	Attenuated *Salmonella Typhimurium*	90
Immunotherapy	RM1, DU145, and PC-3	Probiotics *Akkermansia muciniphila*	91
Immunotherapy	HepG2	Probiotics *Lactobacillus rhamnosus GG*	92
Immunotherapy	B16-F10, MC38, and CT26	*E. coli* DH5α	93
Immunotherapy	B16-F10 and CT26	*E. coli* BL21 (DE3)	94
Immunotherapy	B16-F10 and B16-OVA	*E. coli* TOP 10	95
Immunotherapy with chemotherapy	A549	Attenuated* Klebsiella pneumonia*	96
Immunotherapy with chemotherapy	B16-F10 and 4T1	Attenuated *Salmonella*	97
Immunotherapy with gene therapy	SKOV3, BT474, and HCC-1954	Attenuated* E. coli* W3110△*msbB*	38
Immunotherapy with gene therapy	B16-OVA	Attenuated* E. coli* W3110△*msbB*	98
Immunotherapy with PTT*^d^*	4T1	Attenuated* E. coli* W3110△*msbB*	35
Immunotherapy with PTT	B16-F10	*E. coli* DH5α	99
Immunotherapy with PTT	CT26 and CT26-luc	*E. coli* BL21 (DE3)	100
Immunotherapy with PTT	4T1	Attenuated *Salmonella*	101
Immunotherapy with PTT	CT26 and 4T1	Attenuated* Salmonella typhimurium* △*ppGpp^c^*	102

*^a^nlpI*, encoding lipoprotein NlpI, the deletion of *nlpI* increases the amount of BEVs. *^b^pagP*, encoding Lipid A palmitoyltransferase, which is important for virulence in *E. coli.* *^c^ppGpp*, encoding guanosine 5′-diphosphate-3′-diphosphate. *^d^*PTT, photothermal therapy.

**Table 3:** The major advantages and challenges of BEVs in cancer therapy

Advantages of BEVs	Challenges of BEVs
Intrinsic immunomodulatory properties; Ease of industrialization; Ease of customization; Higher safety.	Lack of standardization; Potential off-target effects; Potential biosafety; Ambiguous contents.

## Abbreviations

OMVs: Outer membrane vesicles
OIMVs: Outer-inner membrane vesicles
EOMVs: Explosive outer-membrane vesicles
EcN: *Escherichia coli* Nissle 1917
TEM: Transmission electron microscopy
NTA: Nanoparticle tracking analysis
WB: Western blotting
PAMPs: Pathogen-associated molecular patterns
PRRs: Pattern recognition receptors
*E. coli*: *Escherichia coli*
PD-L1: Programmed death 1 ligand 1
PD1: Programmed death 1
HA: Hyaluronic acid
Hy: Hyaluronidase
DOX: Doxorubicin
CTLs: Cytotoxic T lymphocytes
KSP: Kinesin spindle protein
HER2: Human epidermal growth factor receptor 2
TSA: Tumor-specific antigens
MHCI: Major histocompatibility antigen complex I
PTT: Photothermal therapy
PTAs: Photothermal transduction agents
NIR: Near-infrared
Mel: Melanin
RBCs: Red blood cells
Mal: Maleimide
1-MT: 1-methyl-tryptophan
ICG: Indocyanine green
NPs: Nanoparticles
CCs: Cancer cells
HPDA: Hollow polydopamine
EPVs: Eukaryotic-prokaryotic vesicles
CCMVs: Cancer cell membrane vesicles
PLGA: Poly (lactic-co-glycolic acid)
FMT: Fecal microbiota transplantation
ctDNA: Circulating tumor cell (CTC), Circulating tumor DNA
